# Strain-level epidemiology of microbial communities and the human microbiome

**DOI:** 10.1186/s13073-020-00765-y

**Published:** 2020-08-13

**Authors:** Yan Yan, Long H. Nguyen, Eric A. Franzosa, Curtis Huttenhower

**Affiliations:** 1 Department of Biostatistics, Harvard T.H. Chan School of Public Health, 677 Huntington Ave, Boston, MA 02115 USA; 2 Broad Institute of MIT and Harvard, Cambridge, MA USA; 3 Division of Gastroenterology, Massachusetts General Hospital and Harvard Medical School, Boston, MA USA; 4 Clinical and Translational Epidemiology Unit, Massachusetts General Hospital and Harvard Medical School, Boston, MA USA

**Keywords:** Microbial strains, Microbial communities, Microbiome, Metagenomics, Amplicons, 16S, Microbiome epidemiology

## Abstract

The biological importance and varied metabolic capabilities of specific microbial strains have long been established in the scientific community. Strains have, in the past, been largely defined and characterized based on microbial isolates. However, the emergence of new technologies and techniques has enabled assessments of their ecology and phenotypes within microbial communities and the human microbiome. While it is now more obvious how pathogenic strain variants are detrimental to human health, the consequences of subtle genetic variation in the microbiome have only recently been exposed. Here, we review the operational definitions of strains (e.g., genetic and structural variants) as they can now be identified from microbial communities using different high-throughput, often culture-independent techniques. We summarize the distribution and diversity of strains across the human body and their emerging links to health maintenance, disease risk and progression, and biochemical responses to perturbations, such as diet or drugs. We list methods for identifying, quantifying, and tracking strains, utilizing high-throughput sequencing along with other molecular and “culturomics” technologies. Finally, we discuss implications of population studies in bridging experimental gaps and leading to a better understanding of the health effects of strains in the human microbiome.

## Background

The importance of phenotypes and physiology characteristic of specific microbial strains has been recognized as early as the nineteenth century. Robert Koch’s postulates, for example, differentiate between disease-causing “pathogens” and benign but closely related microbial variants [1]. While the surprising differences between otherwise similar microbial strains has thus been critical in infectious disease management and microbiology for centuries, it has only recently become accessible in the context of microbial communities and their ecology. It remains technically challenging to detect and differentiate among closely related microbial strains within communities, and we will discuss several high-throughput culture-independent and culture-based methods for doing so here. More importantly, though, the beginning of such work has shown strain variation in the human microbiome to be as important in the structure, function, immunology, and epidemiology of our “normal” microbial residents as it is in the definition of pathogenicity (Box 1).

**Box 1:** Terminology for microbial community strain analysis

Strikingly, there is no universal definition of what constitutes a microbial strain (or, for that matter, species) [2, 3]. Many factors contribute to this difficulty, including the rapidity of microbial evolution, the plasticity of many microbial genomes, the prevalence of mobile elements and lateral transfers, the difficulty in differentiating between many microbial taxa or clades by non-molecular methods, and the overall natural history of microbiology and microbial systematics. This ambiguity has led to a field in which different microbial strains of the same species can differ by as much as 5% nucleotide identity, or 30% or more of their gene content [4]. As such, even apparently benign, phenotypically similar microbial strain variants can differ genomically more than most eukaryotic species, and most related terminology can be context-dependent or defined operationally: **Species:** microbial species have been variously defined based on (1) whole-genome or pangenome nucleotide or amino acid phylogenetic identity thresholds; (2) gross microbial physiology / morphology / phenotype; (3) phenotypes induced by a microbe on its host or environment (e.g., human pathogens); and (4) the host or environment of a microbe, e.g., a specific geographical or biochemical origin [5]. The more than 100-year history of microbial systematics must thus be constantly resolved against new, and emerging, molecular and phenotypic information, leading to operational definitions of microbial species in roughly the two categories of “clades defined as species at some previous point” versus “clades that meet specific quantitative phylogenetic criteria” [6]. These two definitions can be considered roughly equivalent if phylogeny (genotype) is considered to be a trait (i.e. phenotype) by which isolates or community members can be classified into self-similar groups. **Species group or complex:** a group of taxonomically defined species that are not well-differentiated based on genomic or other criteria [7]. These typically arise in microbial systematics due to multiple independent identifications of what later prove to be (essentially) the same organism. Conversely, individual taxonomically defined microbial species can later prove to represent implicit complexes, if they, e.g., are not initially differentiated by physiology but are later found to be molecularly distinct. **Subspecies clade:** in communities, an operationally defined group of related organisms or radius of phylogenetic divergence smaller than, and contained within, a parent species [8]. This allows microbial genotypes within communities to be manipulated independently of their potential systematics, since, e.g., some taxonomically defined species may unintentionally capture widely divergent genotypes (and are thus better described using multiple subspecies clades), while others may prove to be closely related or near-identical (and are thus better described as a single species complex). Historically, subspecies have also referred to phenotypically distinct groups within a species [5], which may or may not be monophyletic. **Isolate:** a presumed clonal strain grown, assayed, and manipulated (presumably) axenically (i.e., in monoculture), typically in vitro, after a process such as streaking and/or colony picking [9]. As per canonical references such as Bergey’s Manual [10], when not defined genomically, isolates have been commonly differentiated based on phenotypes such as morphology; medium specificity; serologic, phage, or bacteriocin sensitivity; biochemical reactions; pathogenicity; or other microbial physiology. **Strain:** Historically, this has meant a microbial isolate, although the definition is not well-suited to microbial community studies. In this context, the term is used variously to refer to a specific microbial genome or collection of clonally identical cells (i.e., a genotype); one or more colonies (believed to be) derived from the same progenitor cell; or most often, in practice, a collection of cells or genomes within a relatively small range of phylogenetic variation (i.e., a very narrow subspecies clade).	

Particularly within communities that are by definition collections of heterogeneous cells, it has proven to be technically challenging to detect and differentiate among cells containing such closely related but highly variable genomes. Indeed, it is not yet clear how clonally most microbial lineages remain within typical in vivo communities. This suggests both basic questions about the generation and maintenance of closely related genome variants in any microbial community, and also pressing translational questions regarding the personalization and health consequences of strains in the human microbiome. Because of the extensive genetic and genomic (i.e., functional) differences between even closely related microbial strains, work to date has only rarely been powered to associate “commensal” microbial strains with their health consequences [11–14]. Here, we thus review the ecology and effects known to date for microbial strain variants carried within the human microbiome, quantitative methods for their detection and epidemiology, and potential next steps including characterization of their surprisingly large pangenomic content of biochemical dark matter.

## Unexpected microbial strain diversity in health and disease from population-scale investigations of the human microbiome

Culture-based comparative genetics of isolates has been a mainstay of microbial characterization for decades, and along with culture-independent techniques, it is increasingly important in an era of high-throughput “culturomics” and creative isolation methods [15, 16]. Especially for human pathogens that are both of clinical interest and relatively easily culturable, hundreds or thousands of genomes have been used in some cases to compare strains and their transmission, associate SNV and structural variation to microbial or host phenotype, and define the genetic and evolutionary architectures of species and other clades [17–19]. Metagenomic methods have the unique ability to extend these strain-specific investigations to almost any environment or microbe, while leveraging the insights already built up using isolate genomics. In particular, if a “strain” is considered to be a clonal genotype, it must correspond to a specific set of genes and resulting functionality. This functional perspective on strains has captured a wide range of operational architectures, since some processes are well-conserved across entire clades (e.g., butyrate production in *Faecalibacterium prausnitzii* [20, 21]). Others, conversely, are highly variable even within specific benign or pathogenic species—*Escherichia coli* in the gut being the most prominent example [22].

### Strains in the human gut microbiome

The gut is the greatest reservoir of biomass in the human microbiome, the body’s largest immune exposure, the most well-studied contributor to microbiome-linked disease, and one of the most ecologically diverse human-associated microbial habitats [23]. It is also the source of several of the most canonical examples of radically different microbial physiology among closely related strains, such as the benign *E. coli* variants carried in most guts as compared to acute pathogens such as enterohemorrhagic *E. coli* (EHEC) O157:H7 [24], long-term risks such as colorectal cancer in association with colibactin production in pks + *E. coli* [25], or the probiotic *E. coli* Nissle 1917 [26]. Isolate cultures have identified other strain-specific characteristics associated with evolutionary advantages ranging from increased virulence [27], mobility [28], nutrient acquisition, antibiotic resistance [29], and defense [30].

Strains abundant in the infant gut are only rarely abundant in maternal microbiomes [31–34] and are often replaced within the first 1–2 years of life [35, 36]. Their similarity to maternal, familial, or generally environmental strains is also itself highly variable and species-specific [31, 32, 37], but even small structural variants may be crucial in immune programming during temporally specific developmental windows [38–41]. Like developmental variants of human gene products, such as hemoglobin forms [42], this dynamism in early life has functional consequences: *Bifidobacterium longum*, for example, is selected for human milk oligosaccharide (HMO) utilization [43] in breastfeeding infants, whereas closely related *B. longum* strains in the adult gut frequently possess the capacity to ferment carbohydrates, but not HMOs [44]. Strains abundant in the infant gut are only rarely abundant in maternal microbiomes [31–34] and are often replaced within the first 1–2 years of life [35, 45], but even small structural variants may be crucial in immune programming during temporally specific developmental windows [38–41]. Ultimately, microbial strain variants affect not only host and individual microbes’ physiology, but also the ecology and phylogenetics of the overall gut community: *Helicobacter pylori* is one of the best-known examples of resident microbial genetic variation paralleling that of human host populations [46], but this has recently been shown to be the case for multiple subsets of the gut microbiome, such as *Prevotella copri* [12] or *Eubacterium rectale* [47]. This leads to linkages between the evolution and diversification of gut microbial community strains and host migration, geography, and lifestyle [8, 48].

One of the most crucial environmental factors related to this in the gut is diet, both acutely and over evolutionary time scales. However, the specifics of this relationship have been difficult to tease apart in human populations, due to the challenges of measuring diverse human diets, the confounding of long-term diet with other environmental factors, and the complexity of diet-microbial biochemical interactions. Indeed, diet represents only one aspect of gut microbial interaction with our biochemical environment, with several examples identified to date of strain-specific metabolism of drugs such as digoxin [49], metformin [50], acetaminophen [51], and potentially many others [52]. With respect to diet itself, De Filippis et al. [53], for example, found a greater abundance of *P. copri* among participants more closely adhering to a Mediterranean-style diet enriched with olive oil, fish, fruits, and vegetables. In contrast, Kovatcheva-Datchary et al. [54] observed that even on the same barley-rich diet, *Prevotella* was only enriched among select participants, potentially in a strain-specific manner. De Filippis et al. [55] later found similar heterogeneity among individuals on low-fat diets. Other examples include strains of short-chain fatty acid (SCFA)-producing bacteria with differential responses to fiber-enriched diets [56, 57]. Perhaps one of the most extreme examples of diet-linked strain specificity in the gut are among probiotic organisms such as *Lactobacillus* and *Bifidobacterium*, for which strains characteristic of fermented foods are highly distinct from those more typically resident in the human gut [58]. The health consequences of probiotics can also be strain-specific dependent either on the strain context of the microbiome being entered [59], or on the strain of the probiotic organisms, e.g., the recently proposed ability of some bifidobacteria to facilitate cancer immunotherapy [60].

### Gut microbiome strains as risk factors in gastrointestinal and systemic disease

While many studies have linked overall microbiome structure or microbial species enrichments to gastrointestinal (GI) or systemic disease, relatively few have identified strain-specific microbial variants associated with these diseases. The inflammatory bowel diseases (IBD) are among the best-studied chronic gastrointestinal conditions with respect to the microbiome, and in IBD, subspecies of *E. coli* and *Ruminococcus gnavus* have each been associated with disease severity [61, 62]. Hall et al. [13] noted a particular subpopulation of *R. gnavus* strains more abundant in the IBD gut, enriched for adaptations to oxidative stress response, adhesion, and the utilization of iron and mucus. *Bacteroides fragilis* strains exhibit divergent behaviors leading to differential IgA induction in mouse models of IBD [63] and have been associated with host immunomodulatory effects in monocolonization [64]. While there are decades of work demonstrating the effects of such variants during animal monocolonization, understanding their effects in the human gut remains challenging, since the equivalent of a human genome-wide association study for most microbial community genetic variants (i.e., those not of very high penetrance) would be challenging, given the degree of multiple hypothesis testing necessary to account for the underlying microbial genetic variability [65, 66].

Studies of systemic disease outside of the gastrointestinal tract have also suggested functional roles for specific gut microbial strains. New-onset rheumatoid arthritis patients appear to be enriched for *P. copri* in the gut in some populations, for example, with evidence that this *P. copri* subset may be functionally or phylogenetically distinct [67]. Obesity and type 2 diabetes (T2D) have shown relatively weak taxonomic or functional shifts in the gut microbiome overall, but again using mice to avoid challenges in human population structure, specific strains of *Akkermansia muciniphila* proved to be causal in alleviating these metabolic conditions [68]. In human subjects, at least one study found SNPs specific to *Bacteroides coprocola* subpopulations within a T2D patient group [69]. More broadly, strain-specific promotion of several SCFA producers, including *Bifidobacterium* spp., *Eubacterium* spp., and *Lactobacillus* spp., was selectively enriched by dietary fiber in a randomized clinical trial, improving T2D parameters [70].

One of the most complex conditions bridging the gut microbiome, gastrointestinal, and systemic health has proven to be cancer. Particularly in colorectal cancer (CRC), specific microbial strain functionality can be readily shown to be locally causal, such as DNA-damaging production of colibactin by pks + *E. coli* as introduced above [71] or *B. fragilis* toxin [72]. Other microbes such as CRC-specific lineages of *Fusobacterium nucleatum* have been identified more recently, with mechanisms such as Fap2-mediated binding to host Gal-GalNAc [73] or immunomodulation via TIGIT [74] mediating both their carcinogenicity and their differentiation from typical oral *F. nucleatum* strains. Other mechanisms of microbial influence on GI or systemic cancer remain less well-understood, with strong evidence of resident microbial effects on immunotherapy responsiveness [75–77], but as yet few strain-specific culprits. Likewise, limited studies have shown intratumoral bacteria within and outside of the colon to be capable of direct metabolism of chemotherapeutics such as gemcitabine [78], with potentially many more such microbe-chemical interactions waiting to be discovered.

### Strain carriage and variation in the body-wide human microbiome

While the strain epidemiology of the gut microbiome is perhaps best developed, similar examples exist of the effects of “commensal” and pathogenic strains throughout the human body habitat. As with the gut, the most extreme examples are those of well-studied pathogens [79], such as resistant variants of *Staphylococcus aureus* in the skin and nasal microbiomes [80]. More recently, combinations of culture-independent and high-throughput culture-based methods have exposed within-subject pathogen evolution over the course of months to years [81]. In these cases, as with pks + *E. coli*, resistance functionality such as *mecA* can be attributed to just one or a few loci that are genetically variable among strains via mobile chromosomal or plasmid-encoded elements [82]. More unexpectedly, however, recent findings have pointed to correspondingly strain-specific interactions with non-pathogenic commensals, such as coporphyrin III production by some *Cutibacterium* (formerly *Propionibacterium*) strains inducing *Staphylococcus* biofilm formation [83]. Indeed, due to their biogeographical heterogeneity relative to the gut, exposed topographical surfaces such as the skin, nasopharynx, and lung are among the few body areas where detailed ecology and persistence of multiple competing strains within an individual has been directly observed [84–86], e.g., among *S. epidermidis* strains in psoriasis [87].

Conversely, deep differentiation of strains within an individual is technically more challenging in the vaginal microbiome. Instead, this environment has revealed extensive subspecies heterogeneity between hosts within the dominant *Lactobacillus* and other species of the vagina, again raising issues regarding the exact definition of strains and species among different microbial clades. Specifically, analysis of the intraspecific diversity of vaginally dominant lactobacilli such as *L. jensenii*, *L. iners*, *L. gasserii*, and *L. crispatus* is complicated by the systematics of the clade, which has been under scrutiny for reorganization based on both isolate and culture-independent genomics [88, 89]. Nevertheless, vaginal *Lactobacillus* and other strains can be reasonably stable within individuals over time [90], with particularly large environmental changes such as pregnancy inducing shifts over the course of gestation [91]. As in the gut, such genetic variation between strains can affect health, such as in the determinants of pathogenicity in *E. coli* causing urinary tract infections [92, 93]. In examples from even more acute infectious disease, strain-specific *Lactobacillus* bioactivity can itself contribute to risk of sexually transmitted infection acquisition such as HIV, both due to direct microbial biochemistry [94] and its effect on host immunity [95].

Finally, oral microbiology has historically provided some of the first and most striking examples of phenotypic heterogeneity between closely related microbial isolates [96–98], and this trend holds true in the era of culture-independent sequencing and whole-community studies as well. Indeed, some of the earliest large population-scale surveys of the microbiome found oral site tropism to be a strong driver of subspecies differentiation [99–101], with stable genetic differences among related microbial colonizers of different surfaces—including different teeth—within the same mouth. These potentially adaptive, highly niche-specific variants have begun to be explored at scale, remaining stable within individual up to hundreds of days within subjects [102], but revealing extensive long-term plasticity between members of clades such as the *Neisseria* [11]. While there is extensive ongoing work regarding the role of overall oral microbial ecology in conditions from periodontitis [103] to pancreatic cancer [104] and heart disease [105], the ecological and genomic diversity of the oral microbiota has led to limited strain-specific associations to date. Several have been suggested for, e.g., *Streptococcus* variants in caries [106] or *F. nucleatum* in association with oral cancer [107]—suggesting intriguing links with its role in CRC. These include sufficient detail to implicate microbial processes such as polyamine biosynthesis, motility and chemotaxis, and immunostimulation (e.g., LPS and flagellar components), but without yet a clear picture of the many possible strains across which these functions may be distributed in the complex oral environment.

## Strategies and approaches to identifying community strain diversity

**Fig. 1:**
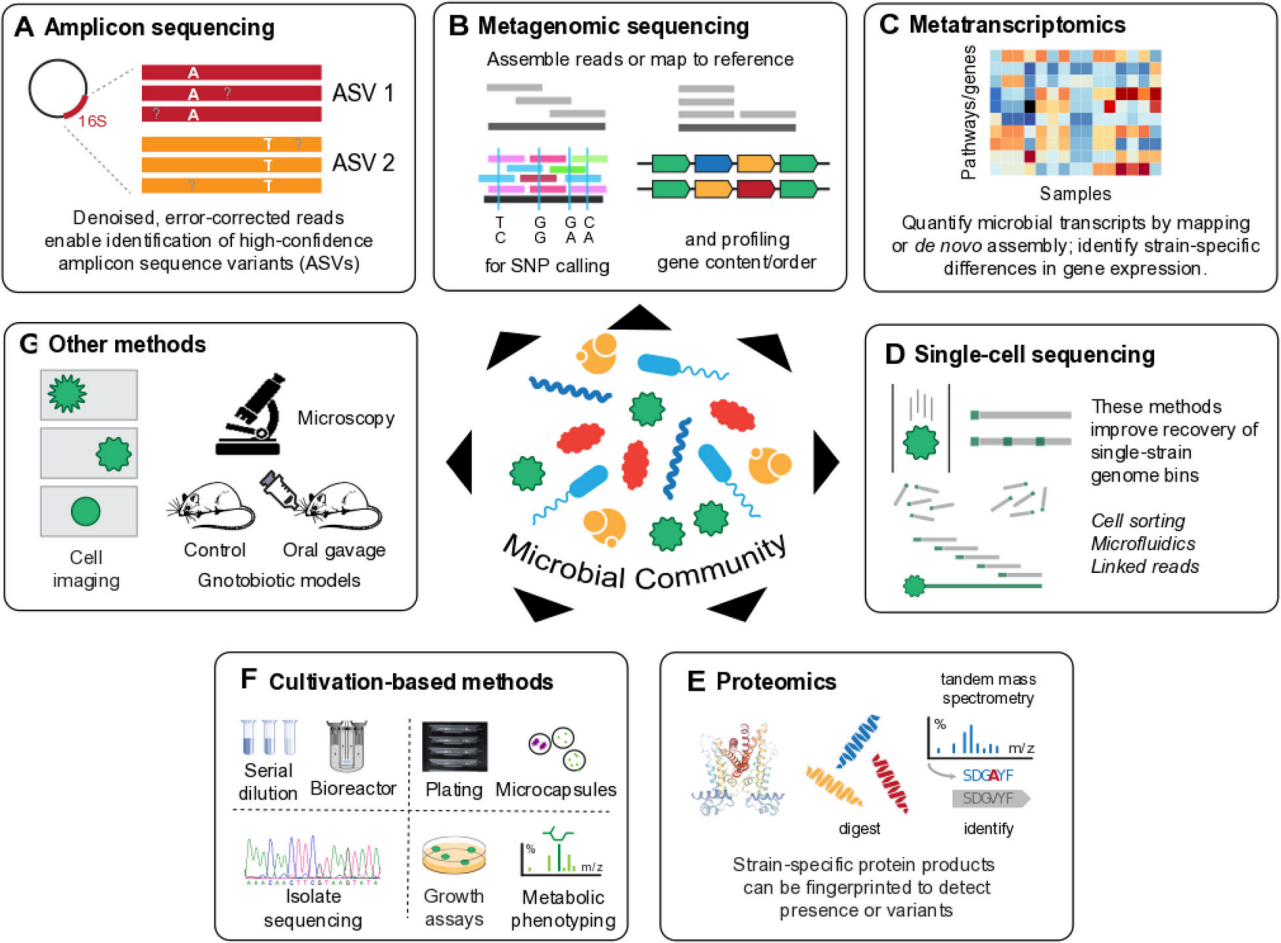
Strain identification approaches for microbial communities. This review summarizes a variety of high-throughput, often (but not always) culture-independent methods for strain identification within microbial communities. **a** Amplicon sequencing (e.g., 16S rRNA gene regions) can now be processed to near-strain-level fidelity, resulting in unique markers such as amplicon sequence variants (ASVs). **b** Shotgun metagenomic sequencing, either via assembly or using reference-based approaches, can identify strains broadly based on their single-nucleotide variants (SNVs) or structural variants (gene gain and loss events). **c** Whole-community transcriptomes can amplify the effects of gene gains or losses, or the effects of small variants that result in differential expression. **d** Single-cell methods can isolate individual microbial genomics directly from within communities, either via cell sorting and amplification, or through synthetic long-read/linked-read techniques. **e** High-throughput “culturomics” can be combined with rapid turnaround approaches such as peptide fingerprinting to strain-type isolates or microcolonies. **f** Relatedly, any combination of traditional isolation and high-throughput cultivation—batch, serial, or continuous—can be combined with growth, phenotypic, or molecular readouts for strain identification. **g** Finally, a variety of other approaches can be used with communities, ranging from flow- or high-content microscopic imaging to systems such as gnotobiotic animal model physiology and phenotyping

It is not our goal here to summarize the many methods that have been used to differentiate among microbial strains in culture over decades of microbiology [108, 109], so we will focus in this review mainly on culture-independent techniques, as well as some high-throughput culture-based methods appropriate for microbial communities (Fig. 1). In both of these categories, many strain definition methods rely on sequencing: assembly of culture-based isolates, or amplicon-based, shotgun metagenomic, or single-cell culture-independent approaches. Other molecular assays, particularly mass spectrometry (MS)-based proteomics, can be applied to strain-type either isolates or communities [110]. This is also true for MS- or NMR-based metabolomics or metabolic flux measurements [111]. Of course, microbial culture physiology and direct imaging has been used to differentiate among strains since the earliest microbiology, and in some cases, these time-tested methods can be applied to communities as well.

### Strain identification from microbial community sequencing

The first breakthroughs in microbial strain identification from whole-community sequencing—like the first community-wide applications of sequencing generally—came from marker gene approaches relying on amplification of 16S rRNA gene variable regions (amplicon or “16S” sequencing, Table 1). In many cases, amplicon-based technologies struggle to differentiate closely related microbial strains, due both to technical (sequencing error, amplification noise, bioinformatics approximations) and biological (lack of nucleotide variants in the amplified regions) limitations [123, 124]. Once data generation platforms reached the fidelity necessary to preserve amplicon biological variation when present, however, several computational approaches emerged to classify such sequences in the most strain-specific manner possible. Oligotyping [125, 126] and Minimum Entropy Decomposition (MED) [114] rely on semi-supervised and unsupervised classification, respectively, of variant positions within otherwise-identical 16S amplicons that show statistically unusual distributions across sample sets (and are thus unlikely due to technical factors). Other types of sub-operational taxonomic unit (OTU) clustering [113] have subsequently extended this intuition to “exact” or “amplicon” sequence variants (ESVs or ASVs, respectively) using statistical error modeling (e.g., DADA2 [115]) or filtering before or after sequence identity clustering (e.g., Deblur [116] or UNOISE2 [117]). Strain-resolved 16S amplicons have been used with methods like these to very specifically link, e.g., *Porphyromonas asaccharolytica* ATCC 25260 and *Parvimonas micra* ATCC 33270 to CRC, for example [127], or to assess the temporal stability of strains in the gut [128]. With additional data generation efforts, they can also generally be extended to multiple -[129] or non-16S amplicons [130], such as the VaST system for identifying a minimum group of target loci for amplification [131]. While SNV diversity in sub-regions of the genome is typically highly correlated with that across the genome [8], the presence or absence of at least one reliably detected SNV within a single amplified 16S variable region can be so precise as to become highly clade- and protocol-specific [115].

**Table 1:** Tools for strain identification in community amplicon and shotgun metagenomic sequencing. Methods and brief summaries of their algorithms for detecting and quantifying strains (by various definitions) from 16S rRNA gene amplicon or shotgun metagenomic sequencing. These are currently the two most prevalent assays for culture-independent strain detection within microbial communities. Note that we have excluded other experimental protocols from this summary, including single-cell, long-read, and synthetic long-read sequencing, since they generally require more than application of a specific software pipeline. These alternatives, and non-sequencing-based approaches, are described in more detail in the text

Method	Platform	Authors’ description	Reference
Oligotyping	16S rRNA gene amplicon	“oligotyping... Focus [es] on the variable sites revealed by the entropy analysis to identify highly refined taxonomic units”	[112]
Sub-OTU clustering	16S rRNA gene amplicon	“we combine error-model-based denoising and systematic cross-sample comparisons to resolve the fine (sub-OTU) structure of moderate-to-high-abundance community members”	[113]
MED	16S rRNA gene amplicon	“MED uses information uncertainty among sequence reads to iteratively decompose a dataset until the maximum entropy criterion is satisfied for each final unit”	[114]
DADA2	16S rRNA gene amplicon	“DADA2 implements a new quality-aware model of Illumina amplicon errors. Sample composition is inferred by dividing amplicon reads into partitions consistent with the error model.”	[115]
Deblur	16S rRNA gene amplicon	“Deblur … compares sequence-to-sequence Hamming distances within a sample to an upper-bound error profile combined with a greedy algorithm to obtain single-nucleotide resolution.”	[116]
UNOISE2	16S rRNA gene amplicon	“UNOISE2... Cluster [s] the unique sequences in the reads. A cluster has a centroid sequence with higher abundance plus similar sequences having lower abundances.”	[117]
PathoScope	Shotgun metagenomic	“PathoID … reassign [s] ambiguously aligned sequencing reads and accurately estimate [s] read proportions from each genome in the sample.”	[118]
LSA	Shotgun metagenomic	“LSA... separates reads into biologically informed partitions and thereby enables assembly of individual genomes.”	[119]
PanPhlAn	Shotgun metagenomic	“PanPhlAn identifies which genes are present or absent within different strains of a species, based on the entire gene set of the species’ pangenome.”	[66]
MetaMLST	Shotgun metagenomic	“MetaMLST performs an in silico consensus sequence reconstruction of the allelic profile of the microbial strains in a metagenomics sample.”	[120]
MIDAS	Shotgun metagenomic	“MIDAS … is a computational pipeline that quantifies bacterial species abundance and intra-species genomic variation from shotgun metagenomes.”	[37]
ConStrains	Shotgun metagenomic	“ConStrains … exploits the polymorphism patterns in a set of universal bacterial and archaeal genes to infer strain-level structures in species populations.”	[121]
StrainPhlAn	Shotgun metagenomic	“StrainPhlAn … is based on reconstructing consensus sequence variants within species-specific marker genes and using them to estimate strain-level phylogenies.”	[8]
metaSNV	Shotgun metagenomic	“metaSNV … performs SNV calling for individual samples and across the whole data set, and generates various statistics for individual species”	[102]
DESMAN	Shotgun metagenomic	“DESMAN identifies variants in core genes and uses co-occurrence across samples to link variants into haplotypes and abundance profiles.”	[122]

Notably, the earliest forms of full-length 16S rRNA gene sequencing avoided many of these issues by capturing biological variation across the entire locus with high fidelity [132], and this has recently become true again in higher throughput with the advancement of “long-read” technologies. Three main platforms can currently provide such long-reads: Pacific Biosciences, Oxford Nanopore, and linked-read analogs such as products from 10X Genomics and Loop Genomics. The extreme fidelity offered by Pacific Biosciences circular consensus sequencing (CCS) has been perhaps best-studied in this context, readily differentiating between single-nucleotide variants (SNVs, although sometimes not insertions or deletions) when they exist anywhere across the 16S rRNA gene locus between strains [133, 134]. Conversely, while Oxford Nanopore’s extremely cost-effective MinION can provide essentially full-length 16S rRNA gene reads, its error rates have restricted strain-specific applications to cases in which no other sequences highly homologous to microbes of interest are present in a community [135–137]. Finally, several protocols now exist facilitating “simulated” long- or linked-reads on a variety of platforms [138, 139], but those which have reached commercial viability are yet to be formally evaluated for amplicon profiling of microbial communities [140]. Similarly, these technologies can sometimes be applied to entire microbial genomes isolated from single cells (e.g., via sorting or microfluidics [48, 141]) or from cross-linked genome copies [138]. This abrogates the need for true metagenomic assembly or binning, as described below, although again with few quantitative studies of these emerging technologies in existence for whole-community profiling at the strain level.

Overall, shotgun metagenomic approaches provide a richer profile of microbial communities’ genetic compositions, as they can in principle identify structural or SNVs anywhere within any microbe’s genome (Table 1). Two broad classes of analyses are currently able to identify microbial strains, the first based on the alignment of metagenomic nucleotides (typically unassembled) to a reference set of genes or genomes. This is generally efficient and sensitive, but of course only possible when sufficiently similar reference genomes (or prior metagenomic assemblies [142–144]) exist to permit direct mapping of metagenomic reads. Notably, “sufficiently similar” references need not be particularly high-identity with respect to a target metagenome. Instead, they must simply permit sufficient genome-wide mapping to identify SNVs or structural variants unique to strains in the community, which can be successful at up to several tens of percent overall nucleotide divergence.

Broadly speaking, four classes of reference-based community strain identification algorithms currently exist. The first identifies the one or more reference genotypes closest to those in a given community, with quantification based on some algorithm for ambiguity-resolved read mapping (e.g., PathoScope [118], Sigma [145]). The second identifies the dominant, potentially novel genotype (strain) per species; these include StrainPhlAn [8], MetaMLST [120], MetaSNV [146], and others [37]. These generally require deeper sequencing (up to 10× or more coverage of the strains to be targeted) and differ in their choice of which reference sequences to map against (e.g., complete genomes vs. universal core genes vs. species-specific marker genes) and the method and stringency of SNV identification. A third class of reference-based methods will further attempt to identify multiple strains per species within a metagenome, such as ConStrains [121] or DESMAN [122], requiring even deeper coverage and more stringent noise removal to prevent false positives. Finally, fourth, methods that rely on structural rather than SNV variants are generally more sensitive (appropriate for community members as rare as ~1× or lower coverage) and include PanPhlan [66] (which can be combined with gene-targeted functional profilers such as HUMAnN [147]), MIDAS [37], and others [4, 65].

Alternatively, when sufficiently similar reference genomes are not available, metagenomic assembly [142–144] can be used for highly novel strain discovery [148]. There is an inherent tension in assembly-based metagenomic strain profiling, as most assemblers seek to identify a single consensus sequence for each contig and require > 1× coverage of an entire genome (or region) to do so. This is appropriate when a single strain dominates its nearby phylogenetic space within a community, in which case less-common strains can be found by mapping metagenomic reads back to, e.g., a binned assembly [149–151] and identifying nucleotide or structural variants roughly as one would within complete genomes [8]. However, in the presence of too many closely related strains within a community, such a consensus sequence is not achievable in the first place, and most assemblers will not be able to provide a contig appropriate for mapping [152, 153]. Even when possible, this process can be further complicated by the high ecological and technical variability of microbial community assemblies, resulting in diverse coverage and confidence (dependent on sequencing depth and population strain admixture) and benefitting from manual inspection of putative variants [154, 155]. Algorithms facilitating this process include Latent Strain Analysis (LSA), which can refine strain-level taxonomy using covariant clusters across multiple related (e.g., longitudinal) samples [132]. Similarly, DESMAN uses statistical models not unlike those for ASV calling in amplicon data to identify variant genotypes well-supported across multiple samples’ co-assembly [122]. In a very few cases to date, strain variants within microbial communities have been identified via analogous differences in metatranscriptomic gene expression quantification, such as strain-specific variation in *Eggerthella lenta* metabolism of the cardiac drug digoxin [49].

**Fig. 2:**
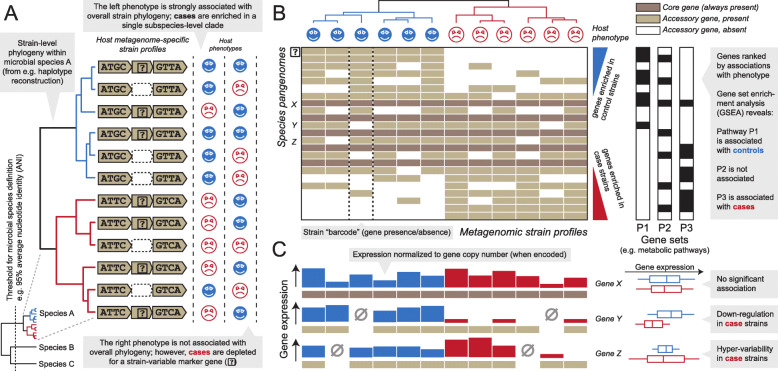
Microbial SNV, structural, and metatranscriptomic variants as features for genetic epidemiology in the human microbiome. Statistical approaches can link subspecies microbial features to human health phenotypes in several ways. **a** When microbial strains are identified using SNV genotypes (whether from genome bins, marker genes, core genes, etc.), any individual microbial SNV—or overall genotype—is typically of low prevalence and high variability. This means that it is extremely difficult to power significant associations with individual SNVs in reasonably sized human population studies. Instead, significant assortment of a host phenotype with strain phylogeny can be assessed, e.g., by PERMANOVA on per-species genetic distances [8] or by aggregating SNVs to genes or larger loci. **b** An extreme of this type of association test directly assesses the nonrandom assortment of genes’ presence or absence among microbial strain pangenomes in association with a phenotype of interest [66], since a gene loss (or gain) is essentially the “sum” of variants at every nucleotide within the gene. **c** Alternatively, even when no differences in genomic SNVs or structural variants are detectable at a study’s level of power, the transcriptional regulatory effects of these variants can be amplified, resulting in strain-specific differences in locus expression in association with a phenotype [156]

Whether from reference sequences or assemblies, SNV versus structural approaches are often complementary and can provide unique information regarding the same underlying community: SNVs (when detectable) identify finer-grained phylogenetic and evolutionary differences, but can be difficult to interpret functionally, whereas structural variants (i.e., gain or loss of full genes or genomic regions) have a lower limit of detection within communities and can speak directly to the biochemical roles of the affected genes (when known, Fig. 2). Unsurprisingly, each approach can provide different strengths and weaknesses. Structural variation can be captured well by reference-based approaches, which are sensitive to unique gene (non-)detection. However, it is very difficult to identify rearrangements (rather than gains or losses) using such techniques, and these are better identified by assembly-based methods instead (when they can be reliably differentiated from, e.g., chimeric assembly errors [157]). Conversely, SNV variation can be well-captured by either reference- or assembly-based approaches—the former more sensitively for organisms with representative isolates, the latter less sensitively but for novel organisms—and by either pangenome or whole-genome mapping approaches, depending where the most uniquely identifying polymorphisms occur. Finally, both structural variation and, to a lesser extent, nucleotide variation are particularly driven in microbial communities by mechanisms of genetic mobility, including all forms of lateral transfer, gene gain/loss, mobile elements, plasmids, and phage integration.

### Other high-throughput molecular methods for strain identification in microbial communities

Other molecular technologies for microbial strain typing in communities are often limited to microbes that can be cultured or otherwise isolated, although advances in (semi-)automated anaerobic culture and nanoculture have made this feasible in high throughput as well. Particularly in clinical microbiology, near-strain variant typing via mass spectrometry peptide fingerprinting is commonplace for pathogen isolates [110, 158], due to its rapid turnaround time and low cost per individual sample relative to sequencing. The technology has some of the same caveats as ASV identification from sequence amplicons introduced above, however: amino acid variants must exist between the strains of interest in the profiled proteins, at a level detectable above experimental noise, and must be classifiable to a taxon of origin in a reference database or by clustering [159, 160]. While in principle the same types of strain-level protein variants could be detected using MALDI-TOF MS technologies in culture-independent community extracts, such applications remain extremely challenging, and instead, community proteomics are currently more commonly analyzed in a gene- or taxon-centric way [161].

Conversely, microbial imaging—arguably the first method for differentiating strains—has made the high-throughput leap to whole communities in several culture-independent forms that are, under appropriate circumstances, able to provide strain-level identification. In some cases, this can mean literally direct microscopy of microfluidically separated (or nanocultured) cells, using automated cell isolation and image analysis [162]. More molecular techniques include spectral or combinatorial fluorescent in situ hybridization (Combinatorial Labeling and Spectral Imaging or CLASI-FISH), which can currently identify over a dozen microbes within a community while maintaining spatial structure [163, 164]. Along with related techniques such as multilabel FISH (MiL-FISH) [165], this relies on the presence of sufficient genetic variants at the FISH-probed loci (often 16S rRNA gene regions) to be differentially bound by spectrally distinct probes, but can in some cases be extended to living bacteria [166]. This is also true for other microbial probe imaging methods such as flow cytometry [167] or light sheet microscopy [168], which can retain viable cells, but require probes or genetically manipulated microbes with loci capable of distinguishing between closely related strains.

While many of these methods are in part or whole culture-independent, it is difficult to understate the importance of the “culturomics” renaissance in separating and characterizing microbial strain isolates from communities including the human microbiome [15, 16, 169]. While pathogen epidemiology has long relied on comparative genomics among up to tens of thousands of isolates, it has only recently become efficient to carry out large-scale isolation of commensal organisms from human populations or individuals [170, 171]. Doing so, however, opens up the ability to identify strain-level differences among isolates of the same species among individuals [12, 13, 172, 173], within an individual microbiome at different spatial locations [81, 174], or over time [170, 175]. Once isolated, of course, such microbial strains can be characterized by any number of standard methods, including differences among growth curves or media, chemical (e.g., antimicrobial) resistance, metabolic flux profiling, or amplicon or shotgun sequencing. Alternatively, whole-community culture via chemostat bioreactors [176] provides an intermediate environment in which strains that are rare in situ can sweep to dominance, or be perturbed in a controlled manner, to amplify differential phenotypes or sequences that may otherwise remain below the limit of detection. Finally, culture-based and culture-independent strain identification techniques blur in the areas of single-cell microbial isolation [177, 178] and microcolony growth [179, 180] from communities. Microfluidic techniques in this vein include gel microdroplets (GMDs) for single-cell amplification [181] or phenotyping [182], as well as microfluidic streak plates (MSPs) [183] that combine the specificity of single cells with the biomass of streaked colonies (if desired).

Particularly when considering culture-based and ex vivo/in vitro/model system assays, the combination of culture-independent high-throughput epidemiology with subsequent strain isolation or manipulation opens up a world of possibilities for characterizing novel health-relevant strains in the human microbiome. This review has taken an essentially “top down” perspective, akin to forward genetics, in which strain-specific features of interest (SNVs, gene cassettes, metabolism, etc.) are identified by various means from human population studies [184]. Such an approach leads naturally to the subsequent biochemical characterization of these variants, either via isolation from primary samples [15, 170] or by in silico retrieval of homologous sequences or related strains from databases or repositories (e.g., ATCC, BEI, DSMZ) [185]. Primary samples can be characterized as an entire community via gnotobiotics [186, 187] or continuous culture [188, 189], or individual isolate strains grown, characterized, or (when possible) genetically manipulated [15, 190, 191]. Such approaches dovetail nicely with “bottom up” approaches (analogous to reverse genetics) that identify and characterize health-relevant strains by directly beginning with isolates and assessing their phenotypes in gnotobiotic mono- or combinatorial colonization [192–197] or, when possible, human feeding [198–200] or microbiota transplant clinical trials [201–205].

## Perspectives and future directions

As introduced above, the precise definition of “strain” is somewhat fluid throughout biology, let alone in microbiology [3] or microbial community biology [206]. While it has most often referred to a single colony isolate culture in the past, the introduction of technologies and tools for precisely resolved genetic variant identification within microbial communities has led to increased broadening of the term. It is now used with some frequency to mean a subspecies or intraspecific clade with relatively low genetic diversity, defined by core or pangenomic identity, nucleotide identity within an amplicon such as the 16S rRNA gene, or the other genotyping or phenotypic similarities described above. As has increasingly been discussed in the literature for microbial systematics overall [8, 207], this suggests the need for a more quantitative definition of strains or subspecies clades, particularly within naturally variant microbial communities. In the absence of a single consensus definition, it is extremely useful for individual studies to define their use of “strain” up front when describing culture-based or (especially) culture-independent microbial community research [174].

Regardless of their precise definition, several emerging technologies offer exciting new approaches for identifying, isolating, and characterizing health-relevant strains in the human microbiome. Historically, microbial genetic variants not associated with an overt, acute phenotype have gone largely undetected, until the relatively recent availability of whole-community profiling techniques by which they can be efficiently captured. Truly single-cell approaches reliant on individual microbial separation have been so far difficult to apply to human epidemiology, with methods for eukaryotic cells not transferring well at scale to the heterogeneity of microbial cell wall biochemistry [208] and methods from environmental community profiling difficult to apply to matrices as diverse as human stool or skin [209]. In addition to bioengineering for cell separation and lysis, advances in low-input, low-noise DNA isolation, amplification, and sequencing will help to address this challenge [210], as will nanoculture approaches that inherently amplify genomes in vivo [180]. Such methods for capturing strains from the human microbiome go hand-in-hand with additional technologies for characterizing them at scale, including cheaper experimental systems such as gut-on-chip [211, 212] or organoid variants [213, 214] that sit in between single isolate culture and rich gnotobiotic models. Ultimately, understanding human microbiome biology will require not just the detection of specific microbial genetic variants in communities, but their introduction and manipulation, including the theoretical ability to genetically perturb any microbial strain either after or even before isolation from its host community [173, 190].

Even in the absence of such technology, extensive work remains to be done to characterize the microbial strain diversity in the human microbiome that has already been uncovered. Of the tens of millions of gene families identified within the human microbiome [23, 99, 215], some ~ 75% are not biochemically characterized by anything more than (in some cases remote) homology to reference sequences, and ~ 25% are not closely homologous to any isolate open reading frames [216]. This astounding pool of biochemical dark matter may be unsurprising to microbial bioprospectors, who have mined primarily environmental communities for novel enzymatic and antimicrobial function for decades [217]. As such, it represents a remarkable potential for new bioactive discovery in human health as well, since human-associated microbes could easily be enriched for protein and metabolite products that modulate host responses [218]. In many of the examples described above, successful associations of SNV or structural variants in the microbiome with human phenotypes or environmental factors have led to genes of unknown function [13, 65, 66]. Strain-level epidemiology in the human microbiome can thus help to prioritize the daunting task of identifying and characterizing the “most interesting” novel microbial variants and products of greatest relevance to health.

Finally, the ways in which better techniques for strain characterization in the microbiome can benefit human health are themselves diverse. Cheap, rapid, and reproducible methods to quantify microbiome SNVs and genetic variants across human populations will allow the identification of precise microbial risk factors, much as did the standardization of human genetics platforms for genome-wide association studies (GWAS) [219]. Also analogously to GWAS, microbial strains can thus provide prognostic or diagnostic biomarkers for disease risk or diagnosis, or hints as to their underlying molecular mechanisms [220–222]. This has been the case for decades in for comparative genetics microbial isolates, and as the number and depth of metagenomes continues to increase, it will undoubtedly become practical in microbial communities as well [223, 224]. Conversely, features of strains found to be bioactive can be used to develop novel interventions for health maintenance or therapy. These can range from better targeting of existing fecal microbiota transplant (FMT) technologies based on donor or recipient strain content [225], to the rational design of synthetic FMTs [226], treatment response prediction for FMTs or prebiotics [227–230], or the eventual administration of genetically modified organisms or communities [231–234]. Recent work in strain-level epidemiology of microbial communities and the human microbiome is thus one of many important, ongoing efforts to realize the microbiome’s substantial translational potential.

## Data Availability

Not applicable.

## Abbreviations

ASV: Amplicon sequence variant
CCS: Circular consensus sequencing
CLASI-FISH: Combinatorial Labeling and Spectral Imaging
CRC: Colorectal cancer
EHEC: Enterohemorrhagic *E. coli*
ESV: Exact sequence variant
FMT: Fecal microbiota transplant
GI: Gastrointestinal
GMDs: Gel microdroplets
GWAS: Genome-wide association studies
HMO: Human milk oligosaccharide
IBD: Inflammatory bowel diseases
LPS: Lipopolysaccharides
MED: Minimum Entropy Decomposition
MS: Mass spectrometry
MSPs: Microfluidic streak plates
OTU: Operational taxonomic unit
SCFA: Short-chain fatty acid
SNP: Single nucleotide polymorphism
SNV: Single-nucleotide variant
T2D: Type 2 diabetes
